# Accumulation and Translocation of Rare Trace Elements in Plants near the Rare Metal Enterprise in the Subarctic

**DOI:** 10.3390/toxics11110898

**Published:** 2023-11-02

**Authors:** Eugenia Krasavtseva, Victoria Maksimova, Marina Slukovskaya, Tatiana Ivanova, Irina Mosendz, Irina Elizarova

**Affiliations:** 1 Laboratory of Nature-Inspired Technologies and Environmental Safety of the Arctic Region, Kola Science Centre, Russian Academy of Sciences, Fersmana St., 14, 184209 Apatity, Russia; v.maksimova@ksc.ru (V.M.); m.slukovskaya@ksc.ru (M.S.); tk.ivanova@ksc.ru (T.I.); ia.mosendz@ksc.ru (I.M.); 2 Institute of North Industrial Ecology Problems, Kola Science Centre, Russian Academy of Sciences, Fersmana St., 14a, 184209 Apatity, Russia; i.elizarova@ksc.ru; 3 I.V. Tananaev Institute of Chemistry and Technology of Rare Elements and Mineral Raw Materials, Kola Science Centre, Russian Academy of Sciences, Fersmana St., 26a, 184209 Apatity, Russia

**Keywords:** rare earth elements, contamination, tailings, *Avenella flexuosa* (L.) Drejer, *Salix* spp., *Betula pubescens* Ehrh

## Abstract

Mining activities create disturbed and polluted areas in which revegetation is complicated, especially in northern areas. For the first time, the state of the ecosystems in the impact zone of tailings formed during the processing of rare earth element deposits in the Subarctic have been studied. This work aimed to reveal aspects of accumulation and translocation of trace and biogenic elements in plants (*Avenella flexuosa* (L.) *Drejer, Salix* sp., and *Betula pubescens* Ehrh.) that are predominantly found in primary ecosystems on the tailings of loparite ores processing. The chemical composition of soil, initial and washed plant samples was analyzed using inductively coupled plasma mass spectrometry. Factor analysis revealed that anthropogenic and biogenic factors affected the plants’ chemical composition. A deficiency of nutrients (Ca, Mg, Mn) in plants growing on tailings was found. The absorption of REE (Ce, La, Sm, Nd) by *A. flexuosa* roots correlated with the soil content of these elements and was maximal in the hydromorphic, which had a high content of organic matter. The content of these elements in leaves in the same site was minimal; the coefficient of REE bioaccumulation was two orders of magnitude less than in the other two sites. The high efficiency of dust capturing and the low translocation coefficient of trace elements allow us to advise *A. flexuosa* for remediation of REE-contained tailings and soils.

## 1. Introduction

For a long time, the mining industry has been one of the major sources of environmental pollution [1,2]. Industrial activity damages ecosystems and threatens human health [3]. Particularly, mining and subsequent waste storage have a complex impact on the environment around the world. Compared to direct emissions, the impact of stored ore processing waste is more complicated and delayed. Tailing ponds harm the ecosystem by spreading pollutants through wind, surface water (including acid mine drainage, i.e., AMD), and groundwater infiltration [4]. This leads to the contamination of soils, plants, and water bodies, the content of potentially toxic elements which far exceeds the natural level [5].

Further, the interaction of pollutants with multielement soil solutions and microbiota changes their mobility, bioavailability, and toxicity [6,7], increasing the contents of pollutants, including rare earth elements (REE), in plants in the vicinity of mining enterprises [8,9]. The chemical composition of plants in impact areas of mining tailings is affected directly by dust deposition from the atmosphere, and indirectly by uptake of chemical elements from contaminated soils [10,11]. The accumulation and integration of pollutants into plant physiological cycles cause a variety of stress reactions, such as a decrease in photosynthetic activity, height, yield, oxidative cellular stress, and many others [12,13,14,15], which consequently lead to the migration of potentially toxic elements through food chains [16,17,18] and cause risks for humans [19,20,21]. Naturally, in the mining zone of REE-containing ores, REE can enter the environment and be accumulated in various ways, creating a potential threat to the ecosystem and public health [22,23]. Despite a growing body of REEs research in the environment, the role of these elements as emerging pollutants and their bio-toxicity remains poorly understood.

Developing effective and sustainable programs for the restoration of industrially disturbed lands to reduce the risk of environmental pollution from decommissioned tailing dumps becomes urgent. It is possible to apply phytoremediation—a nature-inspired approach of sustainable plant cover creation using species resistant to certain pollutants [24]. Phytoremediation can occur in two ways: phytostabilization and phytoextraction [25]. For phytostabilization, plants that accumulate potentially toxic elements in their roots can be used, and for phytoextraction it is recommended to use plants that can absorb and accumulate pollutants from the soil in the aboveground parts [25,26,27]. As seed material, it is recommended to use plants that grow widely in the study area and are resistant to external influences and pollution [4]. Native plant communities growing in metal-contaminated sites can cope with elevated metal levels in soils, so they are much more resilient to this environment than introduced plants (for example, from commercial grass seeds) and can be more effectively used for remediation [28]. Moreover, the use of native plant species to reclaim overburden dumps is a low-cost method that avoids the environmental risks associated with non-native species.

The study of areas of ecosystem self-restoration on tailing dumps is a source of data on the physiological and biochemical mechanisms of plant reactions and the levels of their tolerance to contamination. Thus, a study of false yellowhead (*Dittrichia viscosa* (L.) Greuter) growing on tailings demonstrated an increase in the activity of soluble peroxidases and phenolic compounds, which indirectly indicates the development of a protective mechanism against oxidative stress caused by excess metal content [29]. Alpine fireweed (*Epilobium dodonaei* Vill.) and sand rockcress (*Cardaminopsis arenosa* (L.) Hayek) growing on mining waste also exhibit physiological mechanisms that reduce the harm from increased metal content [30,31]. The results indicate the ability of native plants to adapt to increased content of metals and metalloids in the air and soil, based on individual protective mechanisms. Revegetation is becoming an integral part of mine waste remediation, as plants can accelerate the process of primary soil formation through the production of biomass. However, the effectiveness of this process may vary depending on the type of vegetation.

The current study aimed to fill the gap in the data about the content of REE and nutrients in native plant species growing on revegetated mining tailings at different distances from the dump of the REE enterprise. The largest REE enterprise of loparite ores has been operating in the Russian Subarctic for decades, but we did not find any reports about its effect on the chemical composition of plants in the impact zone. The use of factor analysis and indicators of chemical elements’ accumulation allows us to assess the contribution of an anthropogenic factor in the chemical composition of the aboveground parts of plants. This study provides important information about the suitability of pioneer native species for phytostabilization of the revegetated mining tailings and the subsequent migration of elements along the food chain. The practical goal was to develop a cost-effective remediation strategy for dusted areas in harsh climatic conditions.

## 2. Materials and Methods

### 2.1. Study Area

The study area is located in the vicinity of dusty tailing dumps formed during the beneficiation of loparite ores (Ce,Na,Ca)(Ti,Nb)O_3_. The mining enterprise is located in the center of the Kola peninsula, NE Europe (67.890076° N 34.615571° E), and has been operating since 1951. Two fields of tailing dumps formed during the operation of the enterprise. The first field functioned from 1951 to 1985; since December 1985, tailing pulp has been discharged to the second field of the tailing dump. To date, the total mass of accumulated solid waste exceeds 18 million tons [32]. 

The climatic conditions of the study area are quite harsh. The climate type of the central part of the Kola Peninsula is subarctic continental climate (Dfc according to Köppen climate classification) [33]. In recent years, the territory of the Kola Peninsula has seen an unstable trend towards warmer and longer summers. Further data are provided based on a series of climate measurements from 2012 to 2022 at the Roshydromet (Federal Service of Russia on Hydrometeorology and Monitoring of the Environment) weather station No. 22127, closest to the study area, located in the village of Lovozero [34,35]. The station coordinates are 68.018779 N, 34.99697 E, located at an altitude of 161 m above sea level and at a distance of 20 km in the northeast direction from the sampling points. The average annual air temperature is −1.4 °C, the average annual precipitation is 480.5 mm, with a maximum in August. The following parameters are typical for the sampling period in September. The average daily temperature in September is 9.7 °C, nighttime 4.1 °C. The average wind speed is 3.6 m/s, the average number of rainy days is 13, with precipitation amounting to 23.1 mm. The growing season begins on average on June 10 and lasts for three months until early or mid-September.

The central part of the tailing dump is represented by technogenic sands with a predominance of nepheline, feldspars, aegirine, and sodalite in the mineral composition and the absence of vegetation cover. As we move away from the sand alluvial site, we observe the development of pioneer vegetation groups and a gradual complication of the plant community structure and the formation of an organic soil horizon.

**Figure 1:**
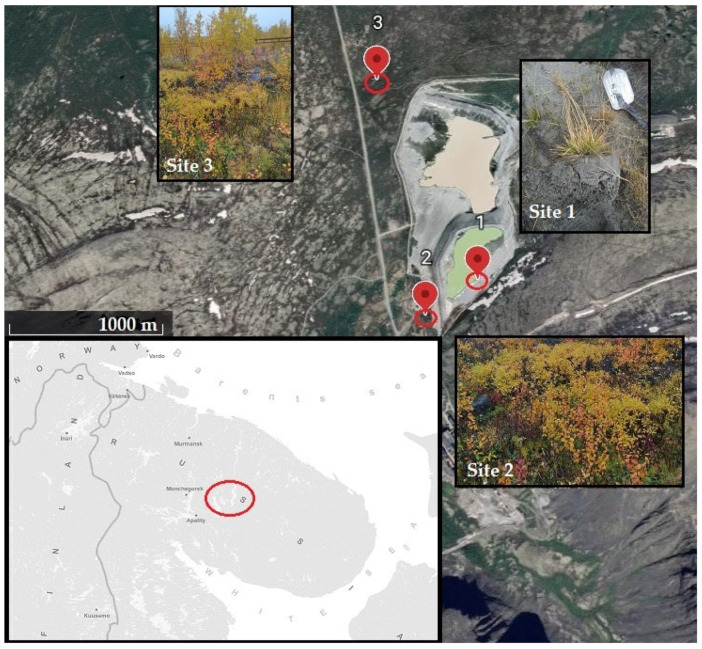
Location and appearance of sampling points.

The studies were carried out on sample plots (10 × 10 m), located in areas of an overgrown tailing dump with varying degrees of development of plant communities and soil profile, as well as a conditional background plot at a distance from the tailing dump (Figure 1).

Site 1 (AV) was located in the area of focal self-overgrowing of tailings sands, 5 m from the upper edge of the pit bowl. Distance from tailing dump was about 0 m. According to WRB 2022 [36], the soils of the first site are classified as Protic Arenosol with technogenic soil formation on nepheline tailing dump and pioneer vegetation. A buried organic horizon [O] was identified in the soil profile, which indicated the re-deposition of nepheline tailing material on the surface after the onset of primary overgrowth processes. The vegetation was represented by a cereal community, with a predominance of *Avenella flexuosa* (L.) Drejer, isolated inclusions of *Salix* sp. and *Betula pubescens* Ehrh., and the dominance of *Polytrichum commune* Hedw. in the moss–lichen layer. The age of the plant community did not exceed 35 years, the mineral composition of the soil was dominated by nepheline, feldspars, and aegirine. 

Site 2 (AO) was located in the south-west direction from the open tailing dump in an area with continuous overgrowth of tailings sands in hydromorphic conditions. Distance from tailing dump was about 100 m. The soil was described as Protic Arenosol also with technogenic soil formation on nepheline tailing dump. The soil profile on this site was highly differentiated from the upper (0–5 cm) layer containing organic material, when the layer 5–10 cm was predominantly mineral. The mineral composition of soil was similar to site 1. The plant community was represented by a grass-forb group of *A. flexuosa* and *Chamaenerion angustifolium* (L.) Scop., dominated by *Salix* sp. and the single presence of *B. pubescens*. and *B. nana* in the tree–shrub layer and the dominance of *P. commune* in the moss–lichen layer. The estimated age of the plant community was 35–70 years.

Site 3 (AP) was located in the north-west direction from the tailings dump and had the zonal soil type—Albic Podzol (Arenic, Folic). Distance from tailing dump was about 300 m. The mineral composition was dominated by quartz, albite, and nepheline. The plant community was represented by a typical shrub moss–lichen community of mountain tundra with the dominance of *B. nana* in the tree–shrub layer; *A. flexuosa* and *Salix* sp. Were presented in single copies.

### 2.2. Sampling and Analysis

Sampling was carried out at the end of the growing season (mid-September). Three plant species were selected at each site: *A. flexuosa* (roots, vegetative aerial parts, ears), *Salix* sp. (leaves), and *B. pubescens* (leaves). Plant sampling was carried out according to the requirements [37]. Sampling of birch and willow leaves was carried out from at least five shrubs for each site. Leaf samples were taken from the upper third of the crown, about 25–30 g from each bush. Wavy hair-grass samples were taken by digging out three (at the tailings site) and five tussocks with plants (at two other sites).

Samples of the upper soil layer (rhizosphere soil) were taken at the sites using the envelope method to a depth of 10 cm to obtain an average sample weighing 2 kg. The pH value of the aqueous extract was determined by the potentiometric method using the I-160MI ion meter. Particle size distribution was analyzed using a Beckman Coulter LS 13 320XR laser (with ALM module and PIDS attachment for measuring particles from 40 nm to 2000 µm, “Beckman Coulter, Inc.”, Brea, CA, USA) particle size analyzer.

Plant samples were washed three times with distilled water. Plant and soil samples were air-dried, ground in agate mortar, and sieved through a sieve with a mesh size of 0.071 mm. 

Soil samples were ground in a powdery state, ashed at a temperature of 550 °C, and an absolutely dry sample was placed in a glassy carbon crucible (SU-2000). The sample decomposition was carried out by the open acid method at a temperature close to the boiling point in hydrofluoric acid during evaporation to wet salts. The sample was then dissolved in concentrated nitric acid while heating, followed by dilution with 2% HNO_3_ while heating. After the sample solution cooled, it was quantitatively transferred into plastic test tubes with screw caps, 4–5 drops of H_2_O_2_ were added and brought to a fixed volume of 20–30 mL 2% HNO_3_ (distilled from “special purity grade”), which was used as a dilution solution and control sample.

Microwave digestion of plants samples was carried out in fluoroplastic autoclaves (Sineo system Jupiter-B series, SINEO Microwave Chemistry Technology (Shanghai) Co., Ltd., China). The sample was ground to a powdery state and the air-dried sample was placed in an autoclave. A mixture of nitric and hydrofluoric acids in a ratio of 16:1 was added to the sample, kept for 8 h, and after the active gas separation stopped, 4–5 drops of H_2_O_2_ were added. The autoclaves were tightly sealed and decomposition was carried out at a temperature of 180 °C. After the sample solution cooled, it was quantitatively transferred into plastic test tubes with screw caps to a fixed volume of 20–30 mL 2% HNO_3_ (distilled from “special purity grade”), which was used as a dilution solution and control sample.

The resulting solutions were analyzed using a mass spectrometer with inductively coupled plasma (ELAN 9000 Perkin Elmer, Waltham, MA, USA) at the Shared Use Center of the Institute of the North Industrial Ecology Problems, Kola Science Center, Russian Academy of Sciences. The quality of analysis was ensured by simultaneous decomposition and analysis of the certified standard samples: birch leaf LB-1 (GSO 8923-2007; SO COOMET 0067-2008-RU), grass mixture TR-1 (GSO 8922-2007; SO COOMET 0066-2008-RU). The observational error was less than 0.5% at *p* = 0.95. The organic carbon content was determined using a CS-2000 Eltra sulfur and carbon analyzer (Eltra GmbH, Hamburg, Germany).

Operational control of the stability of the calibration characteristic was carried out every eight analyzed samples using standard solutions with a concentration in the middle of the calibration characteristic. The drift of the analytical signal did not exceed 2–4%. The precision and repeatability of the analysis were assessed by the standard deviation within 5 h and 5 min, respectively. The value of the standard deviation varied between 2–5%. The accuracy was checked by the degree of similarity of the result of the certified standard samples analysis, the sample preparation of which was carried out in the same way as the analyzed samples. The data obtained were assessed as correct if the analysis result corresponded to the composition of the certified standard samples given in its certificate (passport).

### 2.3. Data Treatment and Statistical Analysis

The content of macro and trace elements in plant samples was compared with the average values of the total element content in *Birch*, *Salix* sp., and *A. flexuosa*, growing in Northern and Eastern Europe [38,39].

The ratios of contents in the initial (not washed) and washed samples were calculated to identify the anthropogenic influence on the chemical composition of aboveground plant organs. Factor analysis was carried out using MS Excel 2016, StatPlus package (version v7, AnalystSoft Inc., Vancouver, BC, Canada). The Kaiser–Meyer–Olkin (KMO) measure of sample adequacy was calculated in R 4.3.1 (R Core Team) using psych package. 

The bioaccumulation coefficient (BC) was calculated as the ratio of the metal concentration in plant tissues to the metal concentration in the soil according to Formula (1):

BC = C*_ip_*/C*_is_*, (1)

where C*_ip_* is the metal concentration in plant tissues (roots, stems, or leaves), and C*_is_* is the metal concentration in the soil [40].

The translocation coefficient (TC) was calculated as the ratio of the concentration in the shoots to the concentration in roots according to Formula (2):

TC = C*_il_*/C*_ir_*, (2)

where C*_il_* is the metal concentration in plant tissues (stems or leaves) and C*_ir_* is the metal concentration in plant roots. TC is a dimensionless coefficient; a higher value implies a higher ability of plants to absorb chemical elements from the soil [41]. Bioaccumulation and translocation coefficients are widely used in modern research to obtain information about the effects of metals on plants, including the assessment of nanoparticles of metal oxides and metal ions in the soil–plant system [42]; studying the accumulation and distribution of metals in plants in areas of industrial pollution [43,44] and the potential of phytoremediation [45].

## 3. Results and Discussion

### 3.1. Chemical Composition of Soils

The content of chemical elements in soil samples is presented in Table 1. The samples were statistically different (*p* < 0.05). The pH value of the water extract was 7.31 ± 0.11, 6.92 ± 0.13 and 6.65 ± 0.10 for samples from areas AV, AO, and AP, respectively. According to the texture (physical clay content (<0.01 mm)), soil samples AV, AO, and AP (8.88, 5.13, and 3.72%, respectively) were classified as sandy soil.

Comparison of the data with the content of REE in background areas of soddy–podzolic soils in the European part of Russia [46] indicates their significant enrichment with elements included in minerals of the developed deposit. This observation was found not only for primary soils, developing on tailing technogenic material, but also on the conditional background soil of the AP site. 

**Table 1:** Content of chemical elements in soil samples.

Element	Tailings [47]	AV	AO	AP	Soils [46]	Soils on Nepheline Tailings [48]
Content, %						
C	0.00	0.27 ± 0.03	4.5 ± 0.31	5.40 ± 0.48	3.5–4.5	8–11
Al	7.86	10.57 ± 0.06	10.34 ± 0.06	14.98 ± 0.06	-	11.53
Ca	-	0.89 ± 0.01	0.76 ± 0.01	2.5 ± 0.06	-	3.87
Fe	3.01	4.4 ± 0.04	3.40 ± 0.07	6.33 ± 0.03	-	6.22
K	-	2.99 ± 0.1	2.49 ± 0.03	3.82 ± 0.04	-	4.51
Mg	-	0.25 ± 0.01	0.21 ± 0	1.24 ± 0.05	-	0.70
Na	-	9.08 ± 0.12	7.09 ± 0.09	6.34 ± 0.06	-	8.61
Si	22.31	22.44 ± 0.36	22.61 ± 0.58	33.02 ± 0.44	-	19.05
Content, mg·kg^−1^						
Ce	852	1813 ± 115	3021 ± 88	264 ± 13	10	388
La	160	933 ± 6	1859 ± 63	167 ± 12	4	279
Mn	1351	1863 ± 57	1411 ± 58	1439 ± 59	-	1394
Nd	106	607 ± 20	962 ± 20	93 ± 2	20	-
Pr	34	223 ± 8	362 ± 18	20 ± 1	1	-
Sc	134	3 ± 1	6 ± 1	4 ± 1	-	-
Sm	12	78 ± 2	121 ± 7	15 ± 1	4	29
Sr	943	1956 ± 199	1511 ± 152	981 ± 20	-	1565
Zn	171	232 ± 8	184 ± 17	195 ± 8	-	113
Zr	2105	2381 ± 66	1269 ± 57	384 ± 8	-	404

Note. A dash means no data.

Comparison of data from the AV site with primary soils formed on apatite–nepheline ores enrichment wastes [48] revealed an increased content of elements included in the loparite ores enrichment tailings, namely: La, Ce, Mn, Si, Sm, Sr, Zr. The content of light group REE (La-Sm) in the AO sample, as well as in the AV sample, exceeded their content in the tailings of the enrichment of loparite ores [47]. Apparently, this is related to the creation and commissioning of the second tailings field, carried out before 1985, and is currently affected by dusting of loose enrichment tailings [49]. Previously, it was shown that finely dispersed tailing material is significantly enriched in the above elements [50]. It should be noted that the REE content in the soils of point AO was higher than AV, which may be due to the peculiarities of REE accumulation by organic matter during the process of soil formation on technogenic parent material under hydromorphic conditions [51]. 

The content of the same elements in the AP soil sample taken at a distance from the tailings dump was significantly lower. In turn, for such basic plant nutrients as Ca and Mg, soil depletion was noted in comparison with Clark values [52] and their contents in primary soils on nepheline sands [48]. It is worth noting that there are quite a few works devoted to the study of the content of REE in the soils of the Kola Peninsula [53,54,55]. Increased REE content was observed in the soils of the area of the largest REE deposit in China—Bayan Obo, which indicated the influence of mining activities on the concentration and distribution of individual REE [56]. A study of the chemical composition of soils near the Bayan Obo mine conducted in 2016 showed anomalous accumulation of REE in surface soils. The average concentration of total REE was 1906.12 mg·kg^−1^, with average values for background soils in China being 181 mg·kg^−1^, varying from 149.75 to 18,891.81 mg·kg^−1^, depending on the sampling site and direction relative to mine. Concentrations of individual light REE exceeded the background values of soils by 20 times for La (518.14 mg·kg^−1^) and Pr (88.81 mg·kg^−1^), 20 times for Ce (982.78 mg·kg^−1^), and 13 times for Nd (262.63 mg·kg^−1^). The order of distribution of average concentrations of individual elements is similar to the distribution in mined ores, which confirms the influence of mining activities on soil composition [56].

Similar studies conducted at the abandoned REE and uranium Mary Kathleen Mine in Central Queensland, Australia [57] showed high soil Ce (1550 mg·kg^−1^), followed by La (645 mg·kg^−1^), whereas the concentrations of Gd (25 mg·kg^−1^) and Lu (1.5 mg·kg^−1^) were significantly lower.

### 3.2. Chemical Composition of Plants

The content of chemical elements in washed plant leaves, as well as the total content of elements in the leaves of *B. pubescens*, *Salix* sp., and *A. flexuosa*, growing in Northern and Eastern Europe, is presented in Table 2, Table 3 and Table 4.

The gross content of Ca, Mg, and, in most cases, Mn in woody plants was lower than the average European background, which indicates that the plant supply by these elements in bioavailable form is scarce. Manganese deficiency has also been noted for wavy hair-grass. At the same time, an aboveground biomass of woody plants contained large amounts of Al and REE, which are part of the enrichment tailings. 

At a similar site, REE concentrations in aboveground and underground parts of plants collected from a site near the inactive Quinta do Bispo uranium mine in Portugal [58] showed accumulation of LREE in both aboveground and underground parts. Thus, for the aboveground part of the *Salix* sp. samples, the REE concentration was 1670 μg·kg^−1^, including LREE—1320 μg·kg^−1^; concentrations of La—349 μg·kg^−1^, Ce—521 μg·kg^−1^, Pr—85.4 μg·kg^−1^, Nd—363 μg·kg^−1^. For the underground part, the REE concentrations were 23,200 μg·kg^−1^, including LREE—19,200 μg·kg^−1^; concentrations of La—5560 μg·kg^−1^, Ce—7640 μg·kg^−1^, Pr—1290 μg·kg^−1^, Nd—4730 μg·kg^−1^. The corresponding content of these elements in the rhizosphere soils of the site amounted to a total of REE—231 mg·kg^−1^, including LREE—195 mg·kg^−1^; concentrations of La—46.9 mg·kg^−1^, Ce—93.0 mg·kg^−1^, Pr—11.5 mg·kg^−1^, Nd—43.3 mg·kg^−1^. According to research [59], in the same areas, the total content of REE in the soil was in the range from 83.6 to 275 mg·kg^−1^, including LREE from 73.9 to 247 mg·kg^−1^.

The wavy hair-grass is characterized by an increased, in comparison with background values, content of Al, Zr, and REE. The absorption of REE (Ce, La, Sm, Nd) by roots correlated with the content of these elements in the soil and was maximum for the AO point. At the same time, the content of these elements in the leaves at this point was minimal, which indicates the activation of biological mechanisms of protection against the toxic effects of high concentrations of REE.

### 3.3. The Anthropogenic Effect on Plants’ Chemical Composition

The chemical composition of aboveground plant parts is affected by the element migration from soil and roots and/or their adsorption from the atmosphere [60,61]. 

The distribution of trace elements and REE was studied in the leaves of some endemic plants, in the atmospheric fallout and soils of rural, urban, and industrial ecosystems in Sicily to indicate the composition of atmospheric dust in [62]. The results of that study confirmed plant exploitation as a bioindicator of environmental quality.

Table 5, Table 6 and Table 7 present the chemical composition of samples of initial aboveground plant parts: leaves of *B. pubescens* and *Salix* sp. and leaves and ears of *A. flexuosa*.

The factor analysis method allows us to determine the level of anthropogenic load on the chemical composition of the study object, and distinguish industrial and natural sources of chemical elements [63,64,65].

**Figure 2:**
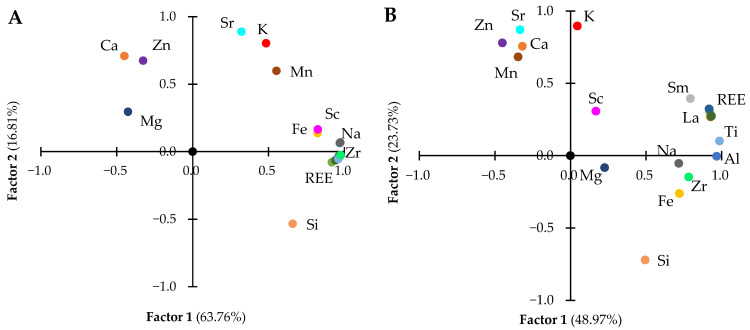
Factor analysis of the chemical composition of the initial (**A**) and washed (**B**) aboveground plant parts. The percentage of variance explained by each factor is shown in the axis labels.

The KMO index verified the sampling adequacy for the analysis. KMO = 0.71, which was well above the accepted limit of 0.5. Based on the results of factor analysis, the two clearest factors affecting the chemical composition of leaves and ears were identified; the contribution of these factors for the initial and washed plant material was 80.6 and 72.7%, respectively (Figure 2).

The first (anthropogenic) factor with a high positive (>0.82) weight included REE, as well as Al, Fe, and Na, present in loparite ores. This factor is most likely associated with the movement of dust flows of solid particles from the tailings pond and their settling on plants. The second (biogenic) factor combined elements such as Ca, K, Mn, Zn, and Sr. It should be noted that the contribution of the anthropogenic factor in the chemical composition of plants after washing decreased by 23% and the contribution of the biogenic factor increased by 29%.

REEs are strongly associated with the mineral matrix and Al_2_O_3,_ TiO_2,_ and Fe_2_O_3_ since they are present in the crystal lattice of minerals in REE deposits [66,67]. At the same time, the REE input to the plant tissues is controlled by the multiply factors such as soil properties, pH, redox potential, root exudates, cation exchange capacity, interactions amongst REEs and compounds, etc. [67]. 

When comparing the results of factor analysis of the chemical composition of the initial and washed aboveground plant parts, an interfactorial transition of some elements was revealed. Thus, such biogenic elements as Zn and Si, after removing surface contamination from the terrestrial parts of plants, were included in the second factor.

**Figure 3:**
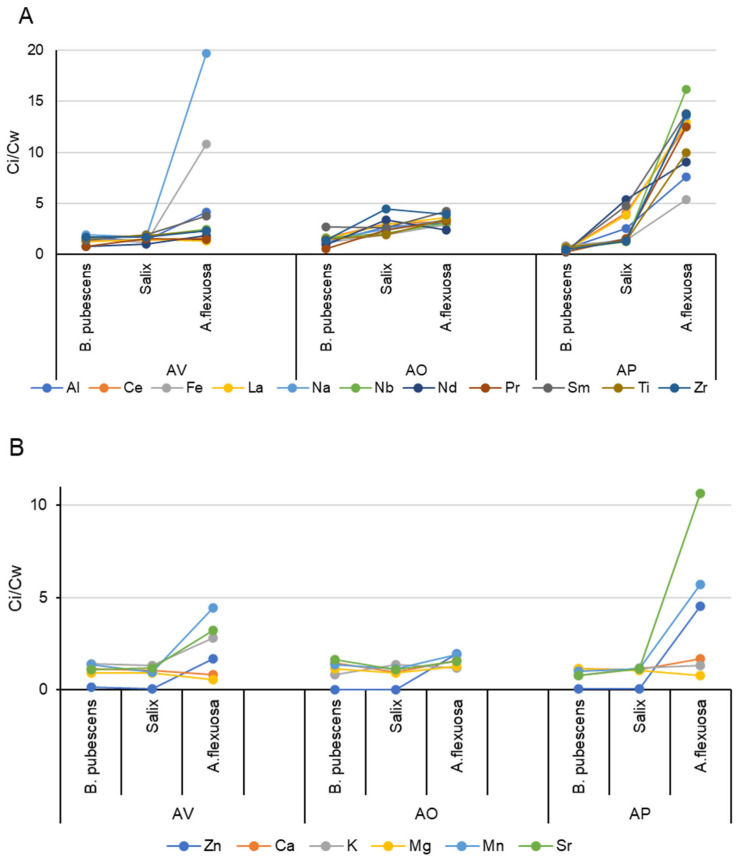
The relationship between the content of elements in the initial (Ci) and washed (Cw) plant material (elements: (**A**) anthropogenic factor, (**B**) biogenic factor).

The relationship between the content of elements in the initial and washed plant material is presented in Figure 3.

The most effective in capturing finely dispersed material among the studied plant species was wavy hair-grass: the proportion of dust deposited on the surface of leaves in areas with maximum anthropogenic load (AV) and with natural soil influenced by dust flows (AP) was the largest for this species. At the same time, in the AO site, which has an average level of dust and the soil of which is composed mainly of tailing material, the contribution of surface pollution to the content of elements was similar for all examined plant species.

The ratio between the content of elements in the initial and washed plant material for biogenic elements in most cases did not exceed 2.0, while for elements with a technogenic source, this ratio was more than 2.0. The exception was birch, for which this ratio in the conditional background area was less than 1.0 and did not exceed 2.0 in other areas.

### 3.4. Bioconcentration and Translocation Coefficients

**Figure 4:**
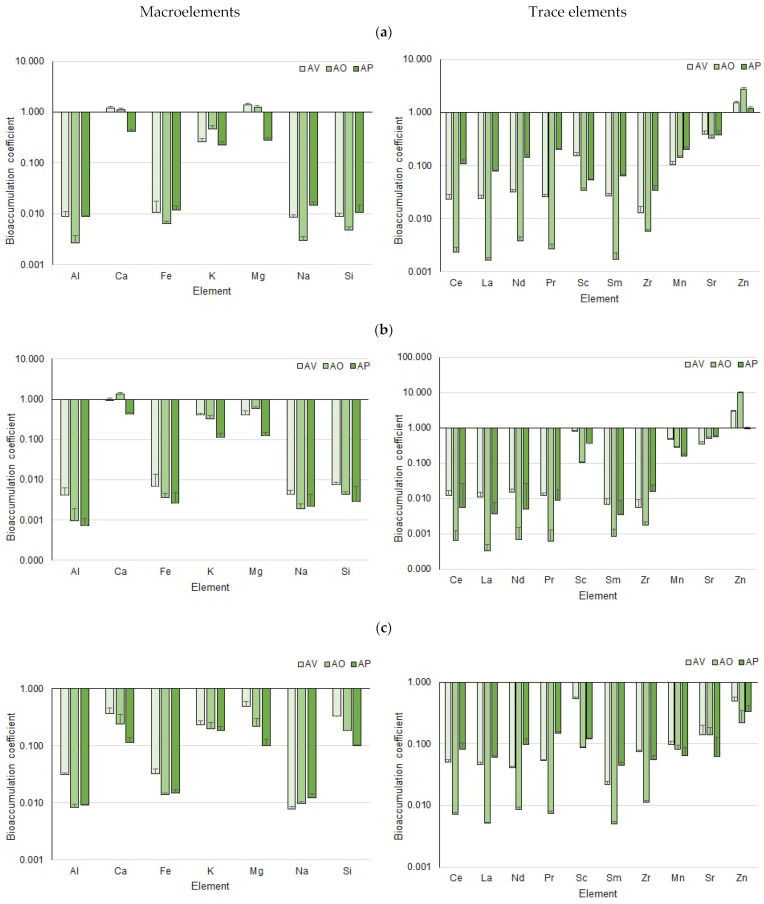
Bioaccumulation coefficients of macro and trace elements in plant samples: (**a**) *B. pubescens*, (**b**) *Salix* sp., (**c**) *A. flexuosa*.

Calculation of bioaccumulation coefficients makes it possible to assess the ability of a particular plant species to increase the accumulation of an element by aboveground plant organs and, accordingly, to remove it from the geochemical cycle, including potentially toxic elements, and also allows you to indirectly assess the supply of soils with certain macrocomponents. Bioaccumulation coefficients of macro and trace elements in plant samples are presented in Figure 4.

Birch accumulated calcium and magnesium, willow accumulated calcium. Both considered species of woody plants accumulated zinc in the aboveground parts of plants: the bioaccumulation coefficient for birch varied from 1.05 to 2.31, for willow from 1.1 to 4.5. The accumulation of zinc in the aboveground parts of willows was also noted in [68]. For wavy hair-grass, all bioaccumulation coefficients did not exceed 1. Noteworthy is the behavior of scandium, whose similarity to the behavior of alkaline earth metals, in contrast to other REE, was noted in [69]. 

Low values of REE bioaccumulation coefficients are probably due to low contents of these elements in mobile, bioavailable forms. Plants containing significantly smaller amounts of REEs compared to soils perform a barrier function in the food chain, preventing the transfer of REEs from the soil to animals and humans, and when absorbed from the soil (as opposed to the aerogenic supply of elements), the maximum accumulation of REEs is noted in the roots of plants [70]. It should be noted that with a higher concentration of REE in the soils of the AO site, the bioaccumulation coefficient in this site was the lowest, which is associated with the conditions of the soil-forming process described above and is consistent with the data obtained in [51].

**Figure 5:**
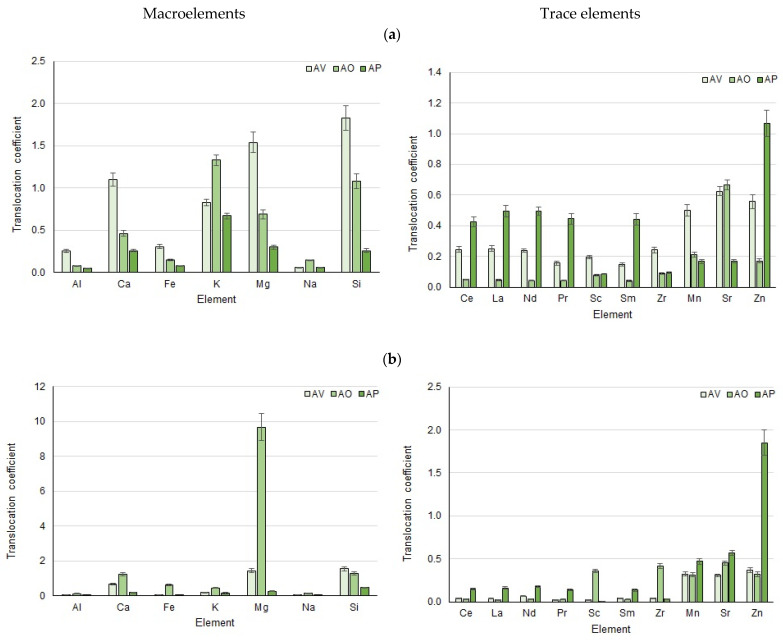
Translocation coefficients for *A. flexuosa* samples: (**a**) leaves/roots, (**b**) ears/roots.

The translocation coefficient was calculated for wavy hair-grass, since only for this plant samples were taken of not only the aboveground biomass (leaves, ears) but also the roots (Figure 5).

There is an active accumulation and transfer of important nutrients: calcium, potassium, magnesium, zinc, and silicon, which are necessary for plant growth. It is reported that under natural conditions, 80% of REE are retained in the roots, and REE concentrations in plant parts decrease in the order of root > stem > leaf > flower > fruit and seeds [71,72,73]. The contents of HM and REE in parts of wavy hair-grass samples were distributed in a similar way: roots > leaves > ears.

High zinc accumulation by aboveground plant parts is explained by the detected increased content of mobile forms in soil samples. As is known, the greatest danger is posed by mobile, bioavailable forms of zinc extracted by ammonium acetate buffer solution [74,75]. The migration of HMs accumulated in the roots to the shoots begins after the roots lose the ability to stabilize or accumulate HMs [76].

The rapid growth of wavy hair-grass and its tolerance to HMs and REEs, as well as its ability to absorb and accumulate metals in the roots, recommend its use for phytostabilization of contaminated soils by limiting the transfer of pollutants further along the food chain [77].

## 4. Conclusions

1. For the first time, a study of three native plants species (*Betula pubescens*, *Salix* sp., and *Avenella flexuosa*) collected in the impact zone of a rare metal enterprise in the Subarctic was carried out. Plants were sampled from sites of partly overgrowing tailings of loparite ores’ processing with varying degrees of soil profile development, as well as from a conditional background area.

2. Analysis of the content of macro and trace elements in soil and plant samples revealed a deficiency of nutrients (Ca, Mg, Mn) at all sites.

3. Two factors affecting the chemical composition of aboveground plant parts were found, the contribution of which for the initial and washed plant material was 80.6 and 72.7%, respectively: anthropogenic (REE, Al, Fe, and Na) and biogenic (Ca, K, Mn, and Sr). The contribution of the anthropogenic factor to the chemical composition of plants after washing decreased by 23%, and the contribution of the biogenic factor increased by 29%. 

4. The REE content in primary soils with a high carbon content (C = 4.5%), formed on tailings under hydromorphic conditions, was higher than in the area with weak development of the plant community on tailings (C = 0.3%) and conditional background soil (C = 5.4%). The absorption of REE (Ce, La, Sm, Nd) by *A. flexuosa* roots correlated with the soil content of these elements. The content of these elements in leaves in the same area was minimal, which indicates the activation of biological mechanisms of protection against the toxic effects of high concentrations of REE. The coefficient of REE bioaccumulation by *A. flexuosa* in this site was in the range of 0.005–0.009, while in the other two sites it was 0.02–0.1. The REE translocation coefficient from roots to leaves and leaves to ears in this site was also an order of magnitude lower than in other sites.

5. The wavy hair-grass *A. flexuosa* is recommended for the phytoremediation of tailings, as it is highly effective in capturing dusty material and has a low content of REE and potentially toxic metals in its aboveground parts, which prevents their migration in food chains. Another undoubted advantage of this species is the ability of the indigenous population to produce a large number of seeds, which makes it possible to carry out reclamation and reduce the damage to public health caused by dusty tailings.

## Figures and Tables

**Table 2:** Content of chemical elements in washed plant samples: *B. pubescens* (leaves).

Element	AV	AO	AP	Birch [38]
Content, %				
Al	0.1 ± 0.001	0.03 ± 0.001	0.13 ± 0.006	0.0027
Ca	1.06 ± 0.02	0.84 ± 0.015	1.06 ± 0.02	1.1
Fe	0.05 ± 0.001	0.02 ± 0.001	0.08 ± 0.001	0.0082
K	0.78 ± 0.01	1.17 ± 0.021	0.87 ± 0.016	1.03
Mg	0.34 ± 0.006	0.27 ± 0.003	0.35 ± 0.004	0.394
Na	0.08 ± 0.001	0.02 ± 0.001	0.09 ± 0.001	<0.002
Si	0.2 ± 0.001	0.11 ± 0.007	0.36 ± 0.003	0.0118
Content, mg·kg^−1^				
Ce	42.68 ± 1.36	6.97 ± 0.33	28.23 ± 1.57	-
La	22.04 ± 1.56	3.11 ± 0.36	13.14 ± 2.67	-
Mn	191.13 ± 2.43	198.16 ± 4.03	285.26 ± 5.08	1470
Nd	19.58 ± 2.53	3.65 ± 0.28	13.07 ± 1.03	-
Pr	5.82 ± 1.76	0.96 ± 0.03	3.86 ± 1.53	-
Sc	0.48 ± 0.13	0.20 ± 0.05	0.23 ± 0.02	<0.03
Sm	2.06 ± 0.29	0.21 ± 0.01	0.95 ± 0.01	-
Sr	755.64 ± 2.13	501.26 ± 6.78	373.94 ± 4.02	36.4
Zn	348.99 ± 1.75	508.67 ± 4.87	229.53 ± 5.03	205
Zr	30.86 ± 0.97	7.00 ± 2.51	28.73 ± 3.06	0.05

Note. A dash means the absence of data in literature.

**Table 3:** Content of chemical elements in washed plant samples: *Salix* sp. (leaves).

Element	AV	AO	AP	*Salix* sp. [38]
Content, %				
Al	0.04 ± 0.001	0.01 ± 0.001	0.01 ± 0.001	0.0025
Ca	0.86 ± 0.016	1.02 ± 0.02	1.09 ± 0.021	1.1
Fe	0.03 ± 0.001	0.01 ± 0.001	0.02 ± 0.001	0.0079
K	1.24 ± 0.028	0.82 ± 0.015	0.43 ± 0.01	1.65
Mg	0.1 ± 0.001	0.12 ± 0.001	0.15 ± 0.001	0.301
Na	0.04 ± 0.001	0.01 ± 0.001	0.01 ± 0.001	0.0023
Si	0.17 ± 0.001	0.1 ± 0.001	0.1 ± 0.001	0.0097
Content, mg·kg^−1^				
Ce	21.44 ± 1.23	1.92 ± 0.12	1.47 ± 0.25	-
La	10.19 ± 1.99	0.61 ± 0.02	0.60 ± 0.01	-
Mn	893.26 ± 4.43	396.57 ± 7.07	225.76 ± 4.83	310
Nd	9.04 ± 1.46	0.66 ± 0.02	0.45 ± 0.01	-
Pr	2.63 ± 1.25	0.22 ± 0.01	0.17 ± 0.01	-
Sc	2.52 ± 1.03	0.61 ± 0.21	1.56 ± 0.74	<0.03
Sm	0.53 ± 0.01	0.10 ± 0.01	0.05 ± 0.01	-
Sr	660.11 ± 7.89	746.40 ± 10.02	536.62 ± 6.49	37.3
Zn	679.44 ± 8.05	1828.24 ± 44.12	183.77 ± 4.38	125
Zr	13.89 ± 3.87	2.24 ± 0.63	4.38 ± 0.03	0.04

Note. A dash means the absence of data in literature.

**Table 4:** Content of chemical elements in washed plant samples: *A. flexuosa*.

Element	AV			AO			AP			*A. flexuosa* [39]
	Roots	Leaves	Ears	Roots	Leaves	Ears	Roots	Leaves	Ears	
Content, %										
Al	1.29 ± 0.03	0.33 ± 0.004	0.05 ± 0.001	1.11 ± 0.006	0.09 ± 0.001	0.15 ± 0.006	2.73 ± 0.029	0.14 ± 0.004	0.03 ± 0	0.0032
Ca	0.3 ± 0.004	0.33 ± 0.004	0.19 ± 0.006	0.4 ± 0.005	0.18 ± 0.006	0.49 ± 0.005	1.11 ± 0.022	0.29 ± 0.003	0.21 ± 0.006	0.1454
Fe	0.47 ± 0.005	0.14 ± 0.001	0.03 ± 0.001	0.32 ± 0.003	0.05 ± 0.001	0.2 ± 0.006	1.22 ± 0.006	0.09 ± 0.001	0.02 ± 0.001	0.0061
K	0.84 ± 0.016	0.7 ± 0.014	0.16 ± 0.004	0.38 ± 0.004	0.5 ± 0.008	0.17 ± 0.004	1.05 ± 0.025	0.7 ± 0.014	0.17 ± 0.004	2.062
Mg	0.08 ± 0.001	0.12 ± 0.001	0.11 ± 0.001	0.07 ± 0.001	0.05 ± 0.001	0.66 ± 0.006	0.41 ± 0.004	0.12 ± 0.001	0.11 ± 0.001	0.1078
Na	1.17 ± 0.025	0.07 ± 0.001	0.05 ± 0.001	0.49 ± 0.005	0.07 ± 0.001	0.07 ± 0.001	1.33 ± 0.03	0.08 ± 0.001	0.03 ± 0.001	0.0014
Si	4.03 ± 0.044	7.39 ± 0.058	6.26 ± 0.058	3.85 ± 0.067	4.17 ± 0.058	4.91 ± 0.058	12.86 ± 0.029	3.33 ± 0.006	6.12 ± 0.031	-
Content, mg·kg^−1^										
Ce	373.74 ± 4.38	91.84 ± 2.94	15.07 ± 1.12	431.55 ± 5.37	21.43 ± 1.23	14.07 ± 1.01	50.80 ± 2.01	21.64 ± 1.92	7.75 ± 2.65	0.05
La	171.99 ± 4.03	43.19 ± 2.97	6.62 ± 1.02	209.99 ± 5.07	9.59 ± 1.87	5.61 ± 1.24	20.75 ± 3.05	10.26 ± 2.25	3.37 ± 1.01	0.027
Mn	361.50 ± 6.87	180.40 ± 4.32	117.79 ± 3.87	546.97 ± 6.93	115.81 ± 3.67	173.07 ± 4.62	551.65 ± 7.03	92.10 ± 2.53	259.48 ± 4.76	529
Nd	104.96 ± 3.08	24.94 ± 3.05	7.06 ± 1.49	199.29 ± 3.97	8.30 ± 1.22	7.37 ± 1.15	18.56 ± 2.41	9.20 ± 1.58	3.37 ± 0.97	0.019
Pr	76.34 ± 4.08	12.00 ± 1.46	2.03 ± 0.69	63.85 ± 5.83	2.69 ± 1.27	1.86 ± 0.98	6.47 ± 4.02	2.88 ± 1.52	0.92 ± 0.05	0.005
Sc	8.81 ± 4.01	1.72 ± 0.97	0.19 ± 0.42	6.43 ± 2.03	0.51 ± 0.21	2.30 ± 0.54	6.18 ± 1.23	0.53 ± 0.04	0.05 ± 0.01	-
Sm	11.70 ± 0.93	1.71 ± 0.05	0.47 ± 0.01	14.48 ± 1.02	0.60 ± 0.02	0.41 ± 0.01	1.53 ± 0.01	0.68 ± 0.01	0.21 ± 0.01	-
Sr	436.09 ± 5.43	272.73 ± 2.09	133.96 ± 2.01	315.42 ± 3.45	210.03 ± 1.84	142.50 ± 2.57	366.67 ± 4.05	62.03 ± 3.02	210.48 ± 1.99	4.2
Zn	202.78 ± 1.78	113.12 ± 1.06	74.11 ± 1.02	236.99 ± 4.07	40.58 ± 7.53	75.73 ± 6.34	60.41 ± 4.65	64.44 ± 3.82	111.88 ± 6.49	28
Zr	334.37 ± 4.08	80.79 ± 7.49	14.99 ± 3.83	229.01 ± 6.45	20.24 ± 3.65	94.53 ± 7.5	227.76 ± 3.35	21.40 ± 4.05	7.29 ± 1.36	-

Note. A dash means the absence of data in literature.

**Table 5:** Content of chemical elements in initial plant samples: *B. pubescens* (leaves).

Element	AV	AO	AP
Content, %			
Al	0.15 ± 0.005	0.03 ± 0.002	0.08 ± 0.002
Ca	1.22 ± 0.03	1.21 ± 0.034	1.21 ± 0.022
Fe	0.07 ± 0.004	0.03 ± 0.002	0.07 ± 0.004
K	1.08 ± 0.013	0.95 ± 0.013	0.66 ± 0.017
Mg	0.31 ± 0.014	0.3 ± 0.009	0.39 ± 0.016
Na	0.15 ± 0.005	0.03 ± 0.002	0.04 ± 0.002
Si	0.22 ± 0.013	0.14 ± 0.005	0.36 ± 0.009
Content, mg·kg^−1^			
Ce	56.60 ± 2.30	11.59 ± 0.59	11.86 ± 0.32
La	28.17 ± 0.37	4.74 ± 0.36	5.08 ± 0.32
Mn	256.58 ± 12.26	269.35 ± 16.47	288.56 ± 16.24
Nd	15.95 ± 0.58	3.33 ± 0.54	3.76 ± 0.31
Pr	4.64 ± 0.38	0.57 ± 0.14	1.02 ± 0.26
Sc	5.46 ± 0.38	0.08 ± 0.01	1.50 ± 0.08
Sm	2.96 ± 0.29	0.57 ± 0.09	0.35 ± 0.05
Sr	803.90 ± 46.21	808.59 ± 32.38	285.35 ± 13.57
Zn	395.20 ± 16.24	700.80 ± 37.15	425.20 ± 17.39
Zr	52.77 ± 2.82	9.99 ± 0.33	13.80 ± 0.43

**Table 6:** Content of chemical elements in initial plant samples: *Salix* sp. (leaves).

Element	AV	AO	AP
Content, %			
Al	0.06 ± 0.006	0.03 ± 0.002	0.03 ± 0.001
Ca	0.89 ± 0.024	0.96 ± 0.015	1.2 ± 0.027
Fe	0.04 ± 0.001	0.02 ± 0.002	0.02 ± 0.001
K	1.62 ± 0.023	1.1 ± 0.029	0.51 ± 0.008
Mg	0.09 ± 0.005	0.11 ± 0.005	0.16 ± 0.006
Na	0.07 ± 0.006	0.03 ± 0.003	0.02 ± 0.003
Si	0.12 ± 0.008	0.3 ± 0.007	0.67 ± 0.013
Content, mg·kg^−1^			
Ce	30.80 ± 1.32	5.82 ± 0.44	5.97 ± 0.57
La	15.17 ± 0.63	1.76 ± 0.32	2.33 ± 0.37
Mn	839.90 ± 33.26	453.75 ± 20.37	259.60 ± 15.46
Nd	9.06 ± 0.70	2.22 ± 0.46	2.45 ± 0.55
Pr	4.03 ± 0.45	0.53 ± 0.10	0.27 ± 0.03
Sc	2.00 ± 0.25	2.00 ± 0.22	2.00 ± 0.15
Sm	1.05 ± 0.28	0.26 ± 0.03	0.24 ± 0.02
Sr	783.51 ± 23.97	793.39 ± 15.26	605.21 ± 13.05
Zn	805.00 ± 29.03	2110.00 ± 55.59	259.60 ± 6.26
Zr	23.69 ± 0.62	9.94 ± 0.26	5.76 ± 0.46

**Table 7:** Content of chemical elements in initial plant samples: *A. flexuosa*.

Element	AV		AO		AP	
	Leaves	Ears	Leaves	Ears	Leaves	Ears
Content, %						
Al	1.39 ± 0.077	0.12 ± 0.007	0.27 ± 0.007	0.06 ± 0.001	1.03 ± 0.012	0.07 ± 0.004
Ca	0.27 ± 0.012	0.24 ± 0.017	0.29 ± 0.008	0.27 ± 0.016	0.49 ± 0.011	0.26 ± 0.012
Fe	1.55 ± 0.022	0.1 ± 0.004	0.14 ± 0.008	0.05 ± 0.003	0.51 ± 0.006	0.07 ± 0.005
K	1.96 ± 0.047	0.21 ± 0.007	0.58 ± 0.009	0.25 ± 0.006	0.92 ± 0.012	0.20 ± 0.017
Mg	0.06 ± 0.004	0.12 ± 0.01	0.06 ± 0.004	0.1 ± 0.006	0.1 ± 0.005	0.11 ± 0.007
Na	1.38 ± 0.032	0.12 ± 0.007	0.24 ± 0.008	0.06 ± 0.002	1.05 ± 0.04	0.07 ± 0.002
Si	15.7 ± 0.443	9.53 ± 0.118	6.66 ± 0.033	10.25 ± 0.061	8.44 ± 0.033	6.86 ± 0.031
Content, mg·kg^−1^						
Ce	153.18 ± 4.21	39.69 ± 1.17	69.40 ± 3.07	15.00 ± 0.58	272.53 ± 9.01	19.21 ± 1.40
La	57.22 ± 1.16	18.00 ± 0.59	34.46 ± 0.40	6.22 ± 0.46	133.30 ± 6.27	8.16 ± 0.37
Mn	801.90 ± 42.33	139.50 ± 5.37	220.22 ± 6.91	291.95 ± 13.69	526.29 ± 20.83	202.44 ± 17.62
Nd	47.23 ± 1.09	10.20 ± 1.02	19.75 ± 0.79	4.55 ± 0.47	83.59 ± 3.79	5.70 ± 1.02
Pr	17.32 ± 0.68	3.48 ± 0.87	8.94 ± 0.31	1.36 ± 0.39	35.93 ± 0.37	1.86 ± 0.08
Sc	124.05 ± 13.02	1.90 ± 0.15	9.23 ± 0.21	0.90 ± 0.27	39.02 ± 1.26	0.08 ± 0.01
Sm	6.41 ± 0.17	1.72 ± 0.28	2.54 ± 0.18	0.50 ± 0.06	9.37 ± 0.32	1.23 ± 0.10
Sr	878.64 ± 37.89	171.59 ± 10.69	320.75 ± 11.78	253.36 ± 12.70	660.95 ± 13.07	186.52 ± 10.90
Zn	185.80 ± 5.95	118.60 ± 4.74	77.24 ± 2.41	148.80 ± 4.79	105.60 ± 4.18	110.60 ± 4.87
Zr	189.60 ± 7.03	29.35 ± 0.78	78.96 ± 1.52	13.58 ± 0.61	293.09 ± 7.78	16.40 ± 0.59

## Data Availability

All data are available within the article.
